# Radioactivity as a driver of bacterial community composition in naturally radioactive mineral springs in the French Massif Central

**DOI:** 10.3389/fmicb.2024.1423342

**Published:** 2024-07-23

**Authors:** Guillaume Holub, Claire Sergeant, Céline Bailly, Aude Beauger, Vincent Breton, Patrick Chardon, Gilles Montavon, Marie-Hélène Vesvres, Clarisse Mallet

**Affiliations:** ^1^University of Bordeaux, CNRS, LP2iB, UMR5797, Gradignan, France; ^2^IMT Atlantique, University of Nantes, CNRS, SUBATECH, UMR 6457, Nantes, France; ^3^University of Clermont Auvergne, CNRS, GEOLAB, UMR6042, Clermont-Ferrand, France; ^4^University of Clermont Auvergne, CNRS, LPC, UMR6533, Aubière, France; ^5^University of Clermont Auvergne, CNRS, LMGE, Aubière, France

**Keywords:** NORM, sediments, radioelements, heavy metals, microbial composition, next generation 16S rRNA sequencing

## Abstract

Some natural environments on Earth are characterised by high levels of radiation, including naturally radioelement enriched mineral springs in the French Massif Central. Therefore, naturally radioactive mineral springs are interesting ecosystems for understanding how bacterial populations in these springs have adapted to high levels of natural and chronic radioactivity over the very long term. The aim of this study was to analyse the bacterial communities of sediments from five naturally radioactive mineral springs in the French Massif Central, sampled in autumn 2019 and spring 2020, and to observe whether radionuclides, compared to other physicochemical parameters, are drivers of the bacterial community structuring in these extreme environments. Physicochemical measurements showed that two springs, Dourioux and Montagne had high radioelement concentrations/activities (uranium, thorium and radon). Analysis of the structure of the bacterial communities, by next generation sequencing based on 16S rRNA gene sequencing, showed that the presence of radionuclides in Dourioux and Montagne, did not lead to a reduction in bacterial diversity and richness compared to the other springs. However, Dourioux and Montagne were characterised by specific bacterial populations, whose presence correlates with the radioelement concentrations/activities measured in these springs. This suggests that radioelements could partly explain the structuring of bacterial communities in these springs. In addition, several of these operational taxonomic units (OTUs) specific to Dourioux and Montagne, mainly affiliated to *Proteobacteria*, *Firmicutes*, *Acidobacteria*, *Actinobacteria*, and *Bacteroidetes*, could be involved in the biogeochemistry of radionuclides through different mechanisms (biosorption, biomineralisation, bioaccumulation, and bioreduction), which would allow the development of other bacterial species sensitive to these metals/radioelements. In particular, the co-occurrence of sulphate and/or iron-reducing bacteria, capable of bioreducing uranium, with fermentative bacteria, releasing sources of organic carbons, reflects associations of bacteria with complementary functions that allow them to grow in this peculiar environment and maintain a high diversity in these extreme environments. This study has provided a better understanding of the structuring of bacterial communities exposed to ionising radiation for thousands of years in naturally radioactive environments.

## 1 Introduction

On Earth, the main source of exposure to ionising radiations is related with natural radioactivity. Indeed, every environmental matrix, whether water, air, soil, rock or living organisms, contains a certain amount of natural radioactivity. This background comes from long-lived radionuclides that appeared during the formation of the Earth, in particular ^238^U, ^232^Th, and ^235^U, naturally present in the Earth’s crust in varying concentrations (Atwood, 2013). In particular, certain environments on Earth display naturally high levels of radiation, known by the acronym NORM (Naturally Occurring Radioactive Materials). In France, the highest concentrations are found in the Massif Central (Auvergne, France), whose bedrock consists mainly of uranium-rich granite (Ielsch et al., 2017; Baker et al., 2023). In particular, the Massif Central is characterised by the presence of mineral springs with high levels of natural radioactivity (NORM; Risler, 1974; Beaucaire et al., 1987; Ielsch et al., 2017). These naturally radioactive mineral springs are defined as isolated ecosystems where concentrations of minerals and radionuclides vary according to many parameters: geological context, water contact time, temperature and solubility of the elements encountered (Fonollosa et al., 2016; Beauger et al., 2021). The radioelements present in these springs come mainly from the chemical alteration of the uranium-rich granitic rocks by groundwater, which will become loaded with ^238^U, ^232^Th, and ^235^U. These isotopes will produce, through spontaneous radioactive decay, descendants that are mostly equally radioactive. In particular, ^226^Ra and ^222^Rn, two alpha particle emitters with a maximum energy of 4.8 and 5.5 MeV, respectively, are found in high concentrations in sediments and mineral spring waters (Sohrabi, 1998; Kitto et al., 2005; Ielsch et al., 2017). These waters then ascend under the effect of hydrostatic or gaseous pressure, with various major and trace elements, until they emerge. Hence, because they are highly mineralised, but also highly radioactive, these springs have specific characteristics for the biodiversity that lives there, including bacterial communities.

Microorganisms including bacteria are the first living things to have appeared on Earth (Djokic et al., 2017; Dodd et al., 2017) and live naturally in complex communities, interacting amongst themselves by exchanging nutrients, competing for substrates and producing chemical compounds. Thanks to their varied metabolic capacities, and their involvement in numerous biogeochemical cycles, bacteria populate many habitats, including the most extreme ones (Falkowski et al., 2008; Gupta et al., 2014; Abatenh et al., 2017). Some of them have the ability to grow in environments with high levels of radiation, without loss of viability and can grow normally under chronic γ-radiation of 50–60 Gy/h, almost one billion times more than the average dose rate on earth (Shuryak and Brenner, 2009; Ragon et al., 2011; Gupta et al., 2014). This resistance to radiation is often associated with genetic modifications that allow better DNA repair, efficient functioning of proteins and other mechanisms necessary for this repair, and also maintenance of cellular homeostasis (Joshi et al., 2004; Daly et al., 2007; Shuryak and Brenner, 2009; Byrne et al., 2014). Bacterial communities regularly encountering high levels of ionising radiation exhibit various adaptive features to support their growth and survival, giving rise to radiotolerant populations specific to such sites (Mondani et al., 2011; Suriya et al., 2017; Chandwadkar et al., 2018; Shuryak, 2019). Hence, high concentrations of radioelements could lead to selective pressure on bacteria, causing native bacterial communities to evolve towards a composition dominated by resistant species, leading to communities that are tolerant to site-specific levels of contamination (Martinez et al., 2006; Mondani et al., 2011; Radeva et al., 2013; Chandwadkar et al., 2018; Hoyos-Hernandez et al., 2019; Jaswal et al., 2019; Rogiers et al., 2021). Several studies have shown that taxa affiliated with *Proteobacteria, Actinobacteria, Bacteroidetes, Firmicutes*, and *Acidobacteria* are dominant in environments contaminated with radioelements and may have specific metabolisms enabling them to resist these high concentrations (Dhal et al., 2011; Islam et al., 2011; Mondani et al., 2011; Kumar et al., 2013; Radeva et al., 2013; Sitte et al., 2015; Yan and Luo, 2015; Suriya et al., 2017; Li et al., 2018; Mumtaz et al., 2018; Jaswal et al., 2019; Rogiers et al., 2021). In particular, these bacterial communities, which are very well adapted to high concentrations of radioelements, can play a major role in the speciation and mobility of radioelements in the environment through various mechanisms (Merroun and Selenska-Pobell, 2008; Shukla et al., 2017; Kolhe et al., 2018; Lopez-Fernandez et al., 2021). Although many studies have characterised the bacterial communities present in environments rich in radioelements due to human activities (former uranium mine or nuclear accident; Elias et al., 2003; Xu et al., 2010; Chapon et al., 2012; Zirnstein et al., 2012; Radeva et al., 2013; Yan and Luo, 2015; Li et al., 2018; Mumtaz et al., 2018; Hoyos-Hernandez et al., 2019; Jaswal et al., 2019), very few have focused on environments where the high radioactivity is of natural origin (Mondani et al., 2011; Nayak et al., 2021). These natural environments, such as mineral springs where radioelements result from alteration of uranium-rich rocks by groundwater, remain underexplored. The study of bacterial populations in naturally radioactive environments allows the analysis of the long-term effects of chronic exposure to radioelements on these communities. This approach provides an advantage for studying long-term effects, in contrast to environments with anthropogenic radioactivity, where bacterial communities are exposed to radioactivity for shorter periods of time.

The naturally radioactive mineral springs located in the French Massif Central are therefore ecosystems where ionising radiation could constitute an abiotic driver influencing the diversity and structuring of bacterial communities. Indeed, the bacterial communities inhabiting these springs have been exposed to high levels of natural and chronic radioactivity for a very long time. As a result, the bacterial communities that develop in these springs have been and are being subjected to selection pressures, resulting from adaptation to these extreme conditions. It therefore seems interesting to study the effects of radioactivity, compared with other physicochemical parameters, on the structuring of bacterial communities in these radioactive mineral springs. To achieve this, we sampled sediments from 5 naturally radioactive mineral springs in the Massif Central (Bard, Dourioux, Graviers, 3 Sauts and Montagne) with varying physicochemical conditions, in autumn 2019 and spring 2020, in order to characterise the bacterial communities using a metabarcoding approach. We focused on the following questions: (i) Is there a difference in bacterial communities between the different springs with varying concentrations of radioelements? (ii) Are these radionuclides, compared to other physicochemical parameters, drivers of the composition of bacterial communities? (iii) Is there a seasonal variation in the structuration of the bacterial communities in these insular mineral springs? Our work covers a wide range of interdisciplinary sciences (biological, chemical, radiological) used to finely characterise these mineral spring samples in order to discriminate the different processes involved in structuring bacterial communities. In addition, to our knowledge, no other study in the literature has characterised extremophile bacterial communities exposed to chronic ionising radiation for several thousand years in mineral springs naturally charged with radioelements, demonstrating the originality of this work. This work is therefore a pioneering attempt to understand how life has adapted to the presence of chronic ionising radiation over long periods.

## 2 Materials and methods

### 2.1 Description of the study site and sampling

**Figure 1:**
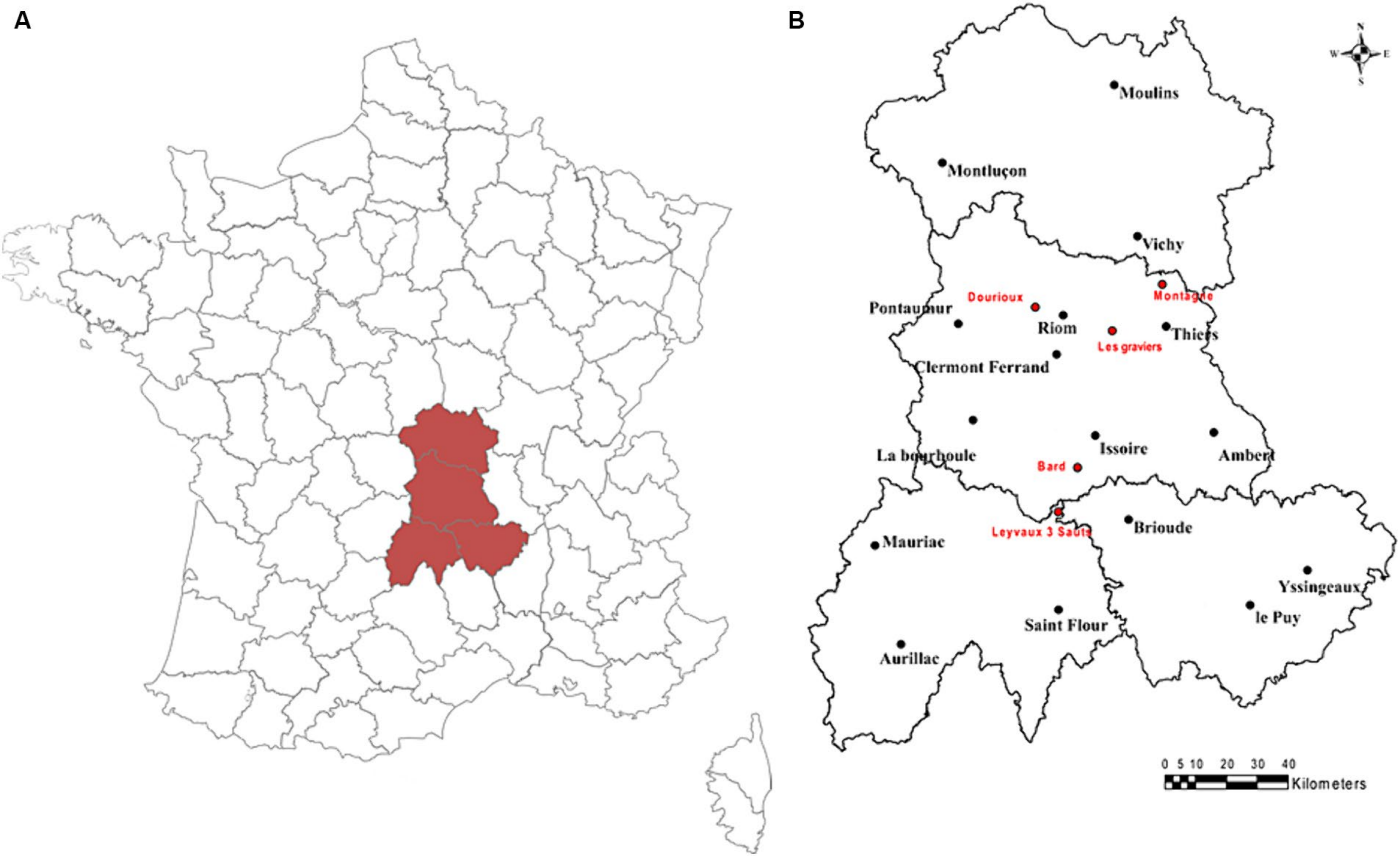
**(A)** Map of France with the location of the Auvergne region; **(B)** Map of the geographical location of the mineral springs sampled in the Auvergne region (France) in October 2019 and June 2020.

For this study, water and sediment samples from five naturally radioactive mineral springs, located in the Massif Central in Auvergne (France), were sampled during two sampling campaigns that occurred in October 2019 and June 2020 (Figure 1). These five mineral springs are 3 Sauts (LE), Bard (B2), Dourioux (DO), Graviers (GR), and Montagne (M1). The main characteristics of these springs are summarised in Table 1. These springs have been selected because they present a significant gradient of physico-chemical conditions, including concentrations of radioelements. Four samples were collected from each spring for the various analyses, three samples for bacterial community analyses (16S rRNA analyses) and one sample for physicochemical parameter analyses. For each spring, the samples were collected on the same day and under the same conditions.

**Table 1:** Location of the 5 springs sampled and description of their main geological characteristics.

Name (acronym)	GPS position	City (department)	Geological characteristics
3 Sauts (LE)	45.3188392°N	Leyvaux (Cantal)	These springs are both located in the Cézallier area, where the bedrock is mainly composed of metamorphic rock. Their chemical composition is rich in chlorine, sodium, bicarbonate, calcium, iron, aluminium, phosphate and trace elements (arsenic, selenium; Fouillac, 1983; Beaucaire et al., 1987; Négrel et al., 2000)
	3.0929669°E		
Bard (B2)	45.4477682°N	Boudes (Puy-De-Dôme)	
	3.1744449°E		
Dourioux (DO)	45.9176246°N	Charbonnières-les-Varennes (Puy-de-Dôme)	This spring takes its source on the western Limagne fault. Surveys had revealed numerous radioactive areas in this region, including the presence of torbernite (Alsac et al., 1988)
	2.9984426°E		
Graviers (GR)	45.8492831°N	Joze (Puy-de-Dôme)	This spring takes its source in the centre of the Limagne fault and emerges from Oligocene sediments. It is rich in bicarbonate, sodium, calcium and magnesium (Risler, 1974)
	3.3136167°E		
Montagne (M1)	45.9836550°N	Châteldon (Puy-de-Dôme)	Rich in bicarbonate and sodium, this spring has its source in the faults on the eastern border of the Limagne. In addition, this spring is known to be one of the most radioactive springs in France (Risler, 1974)
	3.5307306°E		

### 2.2 Analysis of physico-chemical parameters of spring water samples

In order to characterise the physico-chemical profiles of the springs, various parameters were measured in water and sediments. The protocols are described briefly below. More details of the method can be found in Baker et al. (2023).

#### 2.2.1 Spring water samples

Water temperature (°C), conductivity (μS/cm) and dissolved oxygen (%) were measured *in situ* using a multi-parameter probe (WTW FC 340i) and an oxygen probe (Ysi ProODO). Carbonate concentration was measured using a Hach carbonate kit. The concentrations of major elements in the water samples [in mg/L: lithium (Li^+^), sodium (Na^+^), bromine (Br^−^), ammonium (NH_4_^+^), potassium (K^+^), magnesium (Mg^2+^), calcium (Ca^2+^), fluorine (F^−^), chloride (Cl^−^), nitrite (NO_2_^−^), nitrate (NO_3_^−^), phosphate (PO_4_^3−^), and sulphate (SO_4_^2−^)] were measured by a high-pressure ion chromatography technique (Thermo Scientific Dionex Aquion), after filtering the samples (Whatmann GF/C filters). Trace elements [chromium (Cr), manganese (Mn), iron (Fe), nickel (Ni), copper (Cu), arsenic (As), rubidium (Rb), strontium (Sr), caesium (Cs), barium (Ba) and lead (Pb)], as well as radioelements [uranium 238 (^238^U), thorium 232 (^232^Th), radium 226 (^226^Ra)] were also quantified in these water samples, by ICP-QMS (Xseries 2, Thermo Electron) and HR-ICP-MS in low resolution mode (Element XR, Thermo Scientific) with an APEX-Q high sensitivity desolvating sample solution introduction system (only for ^226^Ra) after filtration (0.45 μm PTFE filters—Millipore) and acidification (in 0.65% HNO_3_ medium) of the samples (Subatech, Nantes).

Finally, the activity of radon 222 (^222^Rn) dissolved in water was measured by γ spectroscopy (Bq/L) using a High-Purity Germanium (HPGe) well-type detector (GCW3523, Canberra Inc., Toledo, United States) of 35% relative efficiency. Water was collected according to ISO 5667-1 and ISO 5667-3 standards in Marinelli style gas-analysis containers (NUVIA Instruments GMBH) designed for γ − spectroscopic analysis. The beakers were sealed to avoid any ^222^Rn leakage. ^222^Rn activity was computed under the assumption of secular equilibrium from the 352 keV γ line of ^214^Pb (t_1/2_ = 27.06 min) measured within a few hours after the sampling.

#### 2.2.2 Spring sediment samples

The sediment samples were also chemically and radiologically characterised. Firstly, conventional chemical analyses were carried out to measure the quantities of total organic carbon (TOC in g(C)/kg), total nitrogen (Ntot in g/kg), total hydrogen (Htot in g/kg), total sulphur (Stot in g/kg) and total carbon (Ctot in g/kg) [Cofrac-accredited Inovalys laboratory in Nantes (certificate no. 1–5753 from the French Accreditation Committee)]. For this analysis, the samples were lyophilised (according to standard NF EN ISO 16720) and then sieved to 2 mm. The trace elements (Cr, Mn, Fe, Ni, Cu, As, Rb, Sr, Cs, Ba, Pb, Th, and U) were quantified by ICP-QMS (Xseries 2, Thermo Electron), after mineralisation of the samples and filtration (PTFE filters 0.2 μm; Subatech, Nantes). Finally, the activity of the radioelements (^226^Ra, ^210^Pb, ^212^Pb, ^228^Ac, ^137^Cs) was analysed by gamma spectrometry with coaxial hyperpure Ge detector (GX4519, Mirion, >45% relative efficiency at 1.33 MeV between 10 keV and 10 MeV) according to NF EN ISO 18589-3 (Subatech, Nantes) (National Standards and National Normative Documents, 2015). Measurements were performed at least 4 weeks after the sealing of the sample container to ensure the secular equilibrium between ^226^Ra, ^222^Rn, ^214^Pb, and ^214^Bi. The data were then processed via LabSOCS™ Calibration Software (Mirion Technologies). Sediments were sieved to 2 mm and then dried at 105°C for at least 24 h before analysis following a microwave mineralisation in an acid medium (2 mL HNO_3_ at 15.8 mol/L and 10 mL HCl at 10 mol/L) using a microwave oven (model Ethos Easy, Milestone). The reliability of the results is ensured by participation in a BIPEA interlaboratory test programme for physico-chemical analysis in drinking water and surface water (PT34—Fresh waters—Physico-chemical analyses—Drinking water and surface water) and daily metrological monitoring using a reference solution from this test programme.

### 2.3 Bacterial communities analysis

The composition and diversity of the bacterial communities were analysed using DNA extracts from sediment samples collected from the mineral spring. For each replicate (*n* = 3), an extraction and amplification of the 16S rRNA gene was performed. Each sample weighed approximately 0.3 g. The DNA was extracted using the Fast DNA Spin kit for Soil protocol (MP Biomedicals) according to the manufacturer’s instructions. The extracted DNA fragments were checked by electrophoresis on an 1% agarose gel prepared in 1X TBE (Tris Borate EDTA) buffer containing ethidium bromide (BET). The quantity of DNA in samples was standardised at 30 ng/μL. The following primers were used to amplify the V4-V5 hypervariable region of the gene coding for bacterial 16S rRNA: 515f (5′-GTGYCAGCMGCCGCGGTA-3′) and 928r (5′-CCCCGYCAATTCMTTTRAGT-3′) (Wang and Qian, 2009). Polymerase chain reactions (PCR) were performed in 200 μL microtubes containing a reaction mixture (final volume: 50 μL) consisting of: 30 ng DNA, 10% 10X buffer containing 100 mM Tris–HCl, 500 mM KCl and 0.01% gelatin (Eurobio), 1.5 mM MgCl_2_, 200 μM dNTP (Sigma-Aldrich), 0.4 μM of each primer, 100 μg/mL BSA (bovine serum albumin) and 0.04 unit/μL of Taq DNA polymerase (Eurobiotaq DNA polymerase, Eurobio). PCR cycle parameters were as follows: 1 cycle at 95°C for 5 min (initial denaturation); 35 cycles with 1 min at 95°C (denaturation), 1 min at 55°C (hybridization), and 1 min at 72°C (elongation); and 1 cycle of 10 min at 72°C (termination). PCR amplification was verified by electrophoretic migration of the amplified DNA fragments on a 1% agarose gel. The quantity of DNA was quantified using the Qubit fluorometer with the Qubit dsDNA HS reagent Assay kit (Life Technologies). PCR products were sent to the Get-Plage platform (INRAE, Toulouse, France) for multiplexing, purification and sequencing on the Illumina MiSeq sequencer.

Bacterial sequencing data were analysed via the FROGS (Finding Robust Outlier GeneS) bioinformatics pipeline on the Galaxy platform (Escudié et al., 2018). The FROGS pipeline uses bioinformatics tools to filter, assemble and annotate DNA sequences. Briefly, after a data pre-processing and sequence read assembly step using Vsearch (Rognes et al., 2016), the sequence reads are clustered into operational taxonomic units (OTUs) using Swarm (Mahé et al., 2014), which operates with a clustering threshold of 97%. Finally, after a chimeric sequence removal step and a filtration step (removal of singletons and chloroplast and mitochondrial 16S sequences), the OTUs sequences were compared to the SILVA_132_16s databases (Quast et al., 2013). This bioinformatic analysis resulted in the production of an abundance table of OTUs with their taxonomic affiliations. A pre-filtering step was carried out on the entire table of OTUs for the two sampling campaigns, in order to obtain a more representative table of OTUs for each campaign. This pre-filtering stage consisted of eliminating OTUs whose proportion in all the samples was less than or equal to 0.01% of all the OTUs. This step makes it possible to avoid erroneous results in the subsequent statistical analysis (Lê Cao et al., 2016). Rarefaction analysis was used to check the conformity of the OTU tables. This method is used to assess the diversity within each sample. The rarefaction curves reached a plateau for each sample during the two campaigns, meaning that the number of sequences conserved allowed the majority of phylotypes to be retained. This indicates that the OTU tables after filtering provide a good overview of the overall diversity of the communities studied. Finally, the sequencing data of the bacterial communities were analysed using the phyloseq package of the R software (McMurdie and Holmes, 2013). In particular, this package was used to calculate and graphically display the alpha (bacterial diversity with the Shannon index and species richness with the Chao1 index) and beta diversity indices of the different samples studied. The taxonomic composition of the bacterial communities was also characterised by calculating the relative abundance of each phylum and order for each sample.

All the sequences are available in the Sequence Read Archive (SRA) of NCBI database under the accession number PRJNA1088106.

### 2.4 Statistical analysis

All statistical analyses were performed using R software (version 4.1.1—R Core Team).^1^ Statistical differences between the diversity indices (Shannon and Chao1) of the bacterial communities of each mineral spring studied were analysed by one-way analysis of variance (one-way ANOVA) or Kruskall-Wallis (depending on the conditions of application), followed by a Tukey (for ANOVA) and Dunn (for Kruskall-Wallis) *post hoc* test to observe the statistical differences between each modality in pairs. These tests were performed using the multcom package (Hothorn et al., 2008). A Student’s t-test was performed to compare the means of the diversity indices (Shannon and Chao1) obtained for each sampling period in each mineral spring. The risk α (significance level) was set at 5%. In addition, a Principal Component Analysis (PCA) was performed to observe the potential relationships between the measured physicochemical parameters of the mineral springs and the distribution of the different mineral spring samples according to these measured physicochemical parameters. The PCA analysis was performed using the FactoMineR package (Lê et al., 2008) of the R software. For this analysis, only the elements measured above the detection limits, and for which there were no missing values for each spring during the two campaigns were kept (Supplementary Tables S1A,B). Sparse Partial Least Squares Discriminant Analysis (sPLS-DA) was performed to select discriminant OTUs between different sites using the function ‘splsda’ from R package mixOmics (Rohart et al., 2017). Then Sparse Partial Least Squares (sPLS) was performed using the function ‘spls’ from R package mixOmics (Rohart et al., 2017) in order to observe whether specific OTUs of the different samples were correlated to the physicochemical parameters measured in them. Each of these analyses was carried out for each sample (*n* = 3).

## 3 Results

### 3.1 Characterisation of physical and chemical parameters of mineral springs in October 2019 and June 2020

**Figure 2:**
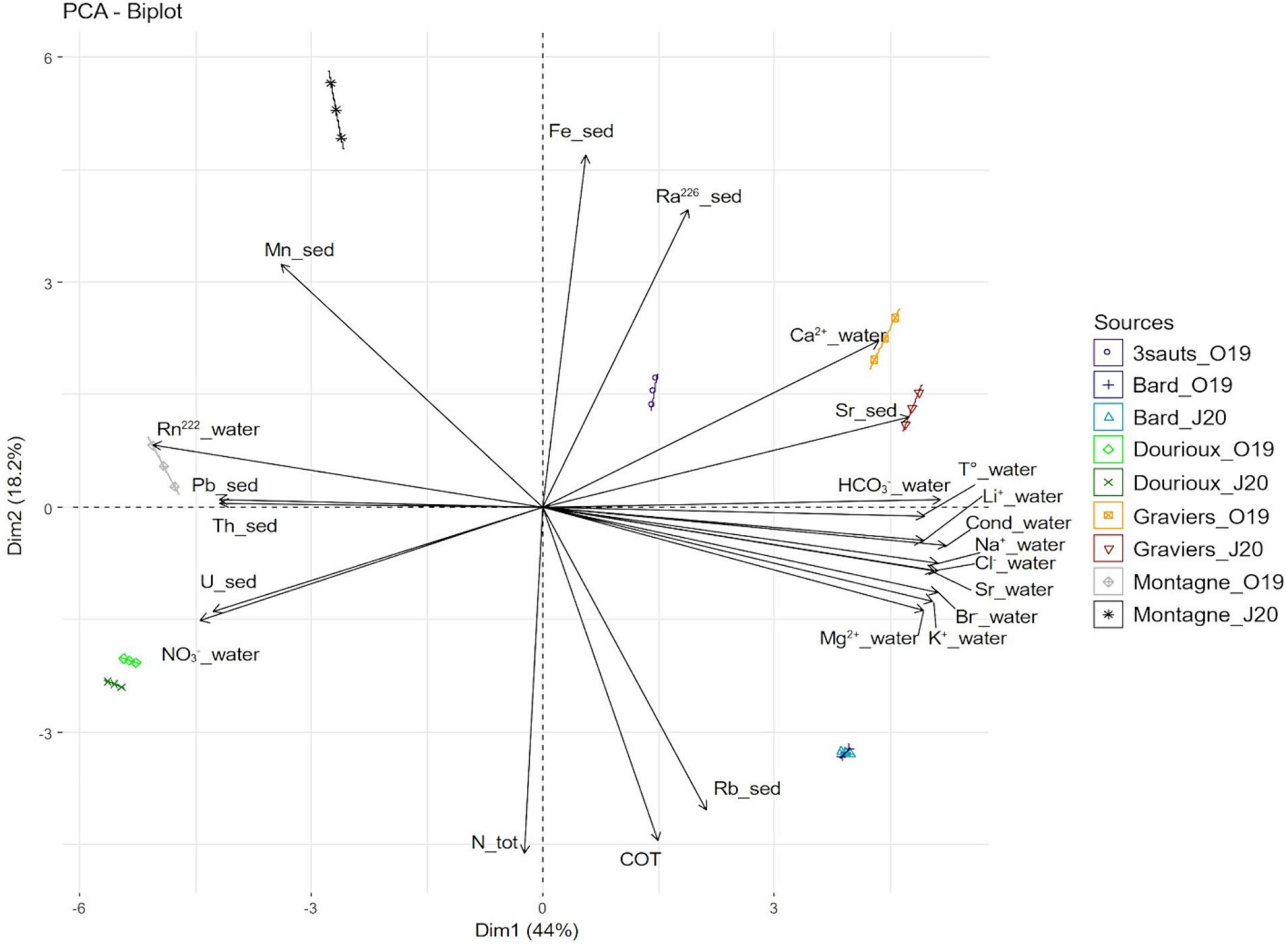
Principal component analysis (PCA) showing the distribution of the springs sampled in October 2019 and June 2020 (except for 3 sauts: no data for June 2020). The analysis is based on 23 physical and chemical variables that significantly contribute to explaining this distribution. The first two axes explain 62.25% of the variance (44.03% for the first axis and 18.22% for the second axis).

The physicochemical measurements, in water and sediment of five springs (Bard, Dourioux, Graviers, 3 Sauts and Montagne) in October 2019, and of four springs (Bard, Dourioux, Graviers and Montagne) in June 2020 are reported in Supplementary Tables S1A,B. The springs are significantly separated from each other in the Principal component analysis (PCA) plot and therefore have different physico-chemical profiles (Figure 2). In particular, Montagne is separated at the top left, Dourioux is separated at the bottom left, Graviers and 3 Sauts are separated at the top right and Bard is separated at the bottom right of the plot. The springs of Montagne and Dourioux are associated with high concentrations of Pb, Th, U and NO_3_^−^, high ^222^Rn activity, low concentrations of major elements (Li^2+^, Na^+^, Cl^−^, HCO_3_^−^, K^+^, Mg^2+^, Br^−^, Ca^2+^), Sr (in water and sediment), and low conductivity and temperature. The Dourioux spring differs from Montagne by a higher concentration of uranium, as well as a high and constant concentration of nitrates during both seasons (Supplementary Tables S1A,B). A variability of physicochemical parameters is observed for Montagne, between the two sampling campaigns. In June 2020, Montagne spring is distinguished by higher concentrations of Mn, Fe, ^226^Ra as well as a lower concentration of nitrates compared to October 2019 (Supplementary Tables S1A,B). On the other hand, the 3 Sauts, Bard and Graviers springs are associated with a high concentration of major elements (Li^2+^, Na^+^, HCO_3_^−^, Cl^−^, K^+^, Mg^2+^, Br^−^, Ca^2+^) and of Sr (water and sediment), high conductivity and temperature, as well as low concentrations of ^222^Rn, U, Th, Pb and nitrates. Graviers spring is characterised by a higher concentration of ^226^Ra, Sr (sediment) and Ca^2+^ whilst the Bard spring stands out with a higher concentration of Ntot, TOC and Rb without seasonal variation.

### 3.2 Bacterial communities diversity and composition in sediment samples

Indigenous bacterial communities in the sediments were identified by sequencing the 16S RNA gene amplicons for a total number of 9,068 OTUs for the October 2019 and June 2020 campaigns. After pre-filtering of the data, 1,438 OTUs were retained for the October 2019 campaign, and 1,366 OTUs for the June 2020 campaign. Measures of alpha diversity, species richness (Chao1) and bacterial diversity (Shannon) are summarised in Table 2. For both sampling campaigns, the measured alpha diversity indices (Shannon and Chao1) were significantly different between the springs studied (*p* value < 0.05). Results show that, during the two campaigns, the alpha diversity (Shannon and Chao1) of the Montagne and Dourioux springs, characterised by higher concentrations of radioelements, was not significantly lower than in the three other springs (Bard, 3 Sauts, and Graviers). For the June 2020 campaign, the Dourioux spring had the highest Shannon diversity index.

**Table 2:** Numbers of OTUs observed, as well as measured alpha diversity indices (Chao1 = richness and Shannon = diversity), in sediment samples from springs sampled in October 2019 and June 2020.

Springs	Observed OTUs		Chao1 (Richness)		Shannon (Diversity)	
	October 2019	June 2020	October 2019	June 2020	October 2019	June 2020
Bard	745 ± 24	686 ± 10	898.29^c^ ± 50.51	837.29^c^ ± 35.75	5.94^c^ ± 0.07	5.72^ab^ ± 0.02
Dourioux	551 ± 13	650 ± 22	658.43^b*^ ± 34.59	732.06^b*^ ± 16.68	5.71^b*^ ± 0.01	5.86^a*^ ± 0.04
Graviers	440 ± 13	451 ± 13	533.85^a^ ± 23.66	542.74^a^ ± 17.68	4.93^a^ ± 0.05	4.88^b^ ± 0.03
3 Sauts	513 ± 15	N.A	640.04^b^ ± 10.51	N.A	4.85^a^ ± 0.01	N.A
Montagne	750 ± 6	626 ± 42	877.26^c*^ ± 26.04	768.04^bc*^ ± 24.34	5.83^bc^ ± 0.04	4.57^ab^ ± 0.95

For diversity indices, data are presented as mean ± standard deviation (*n* = 3). Means followed by the same superscript within a column are not significantly different at *p* < 0.05. Significant differences obtained by a T-test comparison of means between the two seasons are indicated by a * if *p* < 0.05.

**Figure 3:**
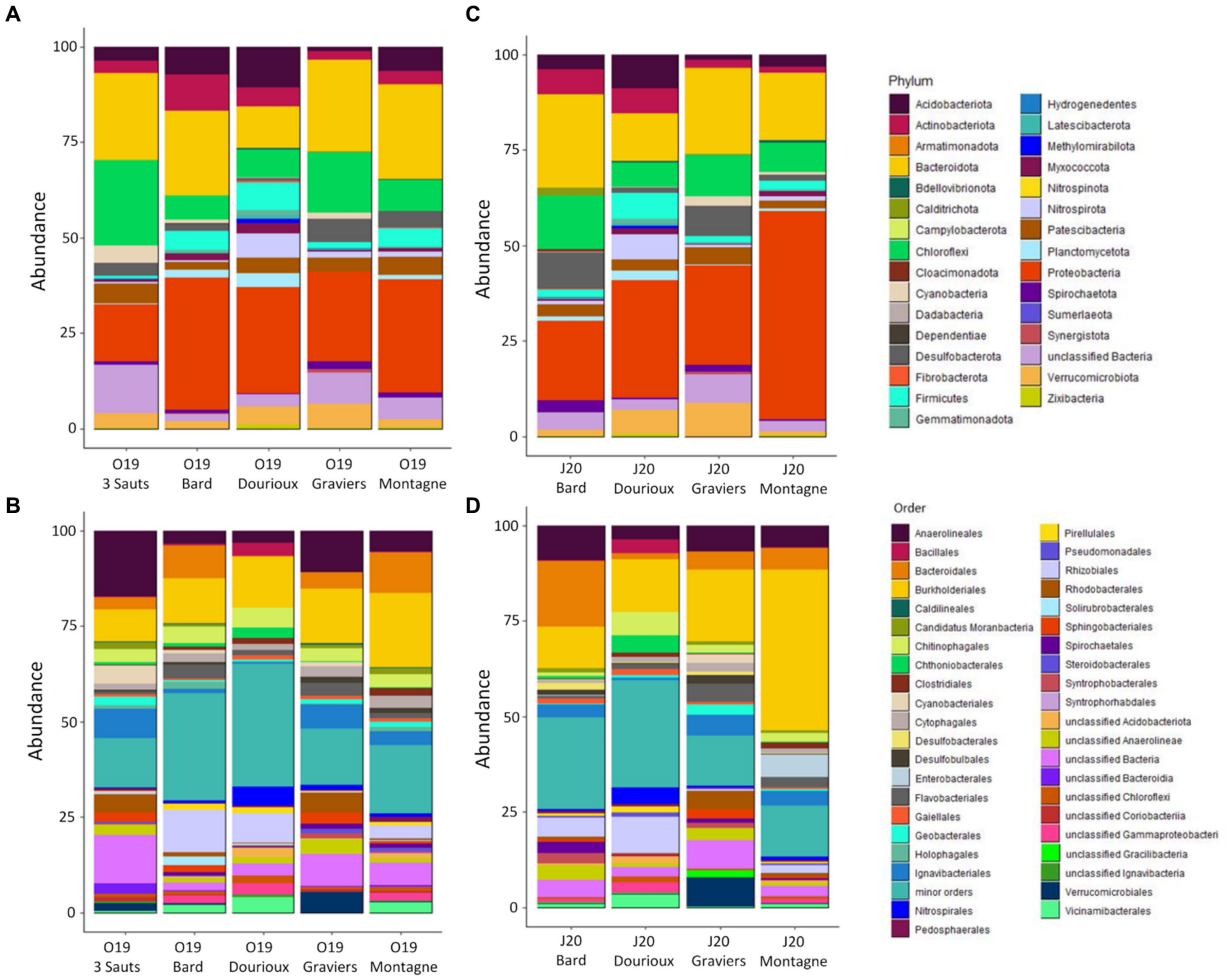
Relative abundance (expressed as % of the total number of OTU reads) of bacteria phyla **(A,C)** and major orders **(B,D)** for the 2019 campaign **(A,B)** and for the 2020 campaign **(C,D)**. Minor orders = orders representing < 0.5% of the total sequences obtained for each campaign.

Analysis of the taxonomic composition of all the spring sediment samples revealed the presence of a total of 28 and 31 different phyla in October 2019 and June 2020, respectively (Figure 3). During the October 2019 and June 2020 campaigns, the majority phyla belonged to *Proteobacteria* (covering between 14.92% in O19 3 Sauts to 54.43% in J20 Montagne), *Bacteroidota* (representing 10.9% in O19 Dourioux to 24.7% in O19 Montagne), *Chloroflexi* (representing 6.2% in O19 Bard to 22.2% in O19 3 Sauts) and *Acidobacteriota* (mostly present in the Dourioux spring:10.6% in O19 and 8.86% in J20; Figures 3A,C). These phyla was, respectively, predominantly represented by the orders *Burkholderiales* (42.1% in J20 Montagne), *Bacteroidales* (17.2% in J20 Bard), *Anaerolineaceae* (mainly in 3 Sauts: 17.3% in O19 and 25.34% in J20) and *Vicinamibacterales* (mainly in Dourioux: 3.4% in O19 and 3.5% in J20; Figures 3B,D). Finally, the phyla *Actinobacteriota, Firmicutes, Verrucomicrobiota, Cyanobacteria, Desulfobacterota, Patescibacteria, Planctomycetota, Nitrospirota, Spirochaetota*, and *Myxococcota* were detected in all samples with average relative abundances higher than 1%, but not exceeding 5% in both seasons. This analysis reveals that the relative abundance of the different phyla and orders identified varies from one spring to another, but also between the two sampling periods for the same spring.

### 3.3 Correlation between spring bacterial communities and their physicochemical parameters

**Figure 4:**
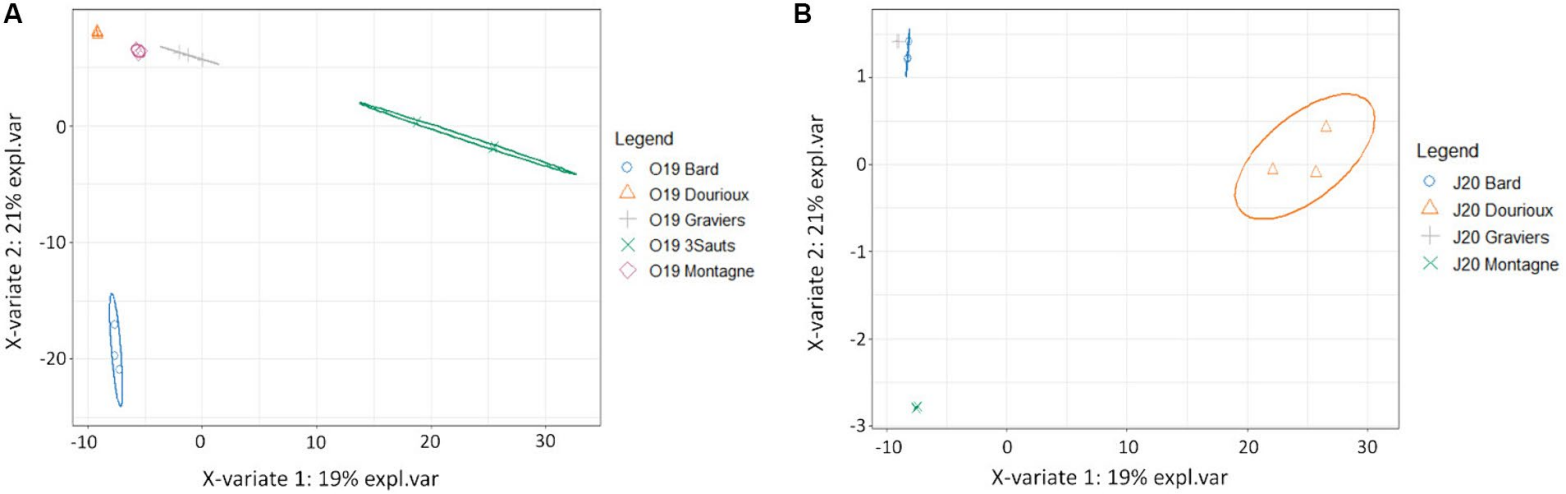
Sample distribution of **(A)** the 2019 **(B)** 2020; campaign due to the predominant OTUs in each, from the sPLS-DA analysis on the first two components with ellipse plots at the 95% confidence level.

Significant changes in bacterial community structure were observed between the sediments of the studied springs in both campaigns due to the presence of specific OTUs in each of them (Figures 4A,B). For the October 2019 campaign, the relative abundance of 54 OTUs made possible the discrimination of Montagne and Dourioux springs, characterised by high concentrations of radioelements (Supplementary Figure S1). On the other hand, the spring of 3 Sauts is discriminated by the presence of 236 specific OTUs and the spring of Bard is discriminated by the presence of 170 predominant OTUs (Supplementary Figure S1). For the 2020 campaign, the springs of Dourioux and Montagne were also discriminated from the other springs due to the presence of specific OTUs in them (Figure 4B). In particular, the spring of Dourioux was discriminated from Montagne, Bard and Graviers, by the presence of 295 OTUs specific to this spring (Supplementary Figure S2). On the other hand, the spring of Montagne was mostly separated from the other 3 springs by the presence of 5 predominant OTUs (Supplementary Figure S2). Finally, Graviers and Bard springs are also separated from the other 2 springs due to the presence of 5 OTUs specific to these springs (Supplementary Figure S2).

**Figure 5:**
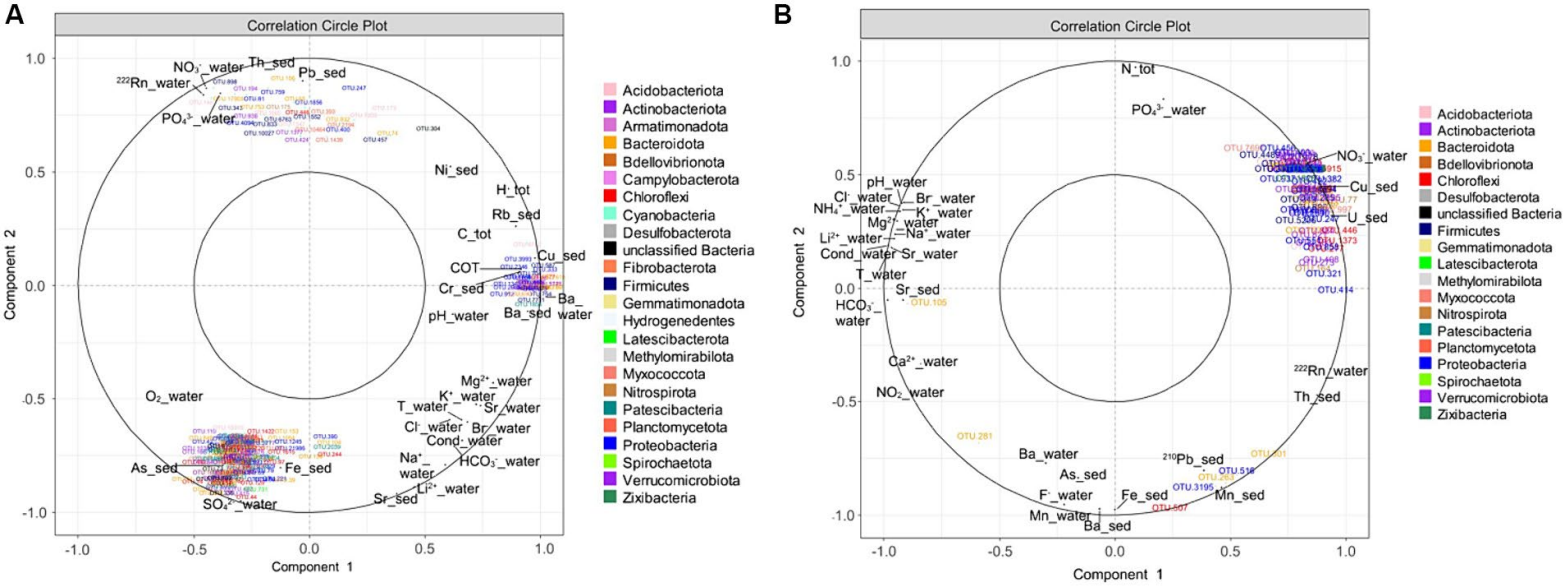
Correlation circle plot of the sPLS analysis highlighting the relationship between specific OTUs and the physicochemical parameters of the sources **(A)** in October 2019; **(B)** in June 2020.

These specific OTUs varied amongst the sampled springs depending on their specific physicochemical properties (Figures 5A,B). For the October 2019 campaign, three clusters of OTUs were identified, each correlated with different physicochemical parameters. The first cluster, in the upper left of Figure 5A, is composed of 35 OTUs specific to the Dourioux and Montagne springs and positively correlated with high concentrations of radioelements and potentially toxic metals (Pb, Th, ^222^Rn), but also with high concentrations of nitrates and phosphates (Figure 5A). These OTUs are also correlated with low concentrations of major ions (Mg^2+^, K^+^, Br^−^, Cl^−^, HCO_3_^−^, Na^+^, Li^2+^), Sr (water and sediment), low temperature and conductivity. These 35 OTUs are affiliated to 10 phyla, including 5 with *Acidobacteriota* (*Vicinamibacterales, Holophagae*); 2 with *Actinobacteriota* (*Arthrobacter* sp., *Streptomyces* sp.); 6 with *Bacteroidota* (*Flavobacterium*, *Chitinophagaceae*); 1 with *Chloroflexi;* 7 with *Firmicutes* (*Paenibacillus, Bacillus, Pelosinus, Desulfosporosinus, Fonticella*); 1 with *Nitrospirota* (*Nitrospira*); 4 with *Planctomycetota* (*Pirellulaceae*); 6 with *Proteobacteria* (*Nitrosomonadaceae, Methylophilaceae, Xanthobacteraceae, Rhodocyclaceae*); 1 with *unclassified Bacteria*; and 2 with *Verrucomicrobiota* (*Chthoniobacteraceae*). In addition, 165 OTUs in the bottom left-hand section of the plot, and specific to the 3 Sauts and Graviers springs, are correlated with high concentrations of metals (As, Fe, Sr), major elements (SO_4_^2−^, Li^2+^), high oxygen content, as well as low concentrations of Th and Pb (Figure 5A). These OTUs were mainly affiliated with *Acidobacteriota* (7 OTUs including *Thermoanaerobaculia* and *Holophagae*); *Actinobacteriota* (9 OTUs including *Solirubrobacterales, Gaiellales* and *Microtrichales*); *Bacteroidota* (28 OTUs including *Bacteroidales*, *Chitinophagales*, *Flavobacteriales*, *Ignavibacteriales* and *Sphingobacteriales*); *Chloroflexi* (28 OTUs including mainly *Anaerolineaceae*); *Patescibacteria* (23 OTUs including *Gracilibacteria, Microgenomatia* and *Parcubacteria*); *Proteobacteria* (23 OTUs including *Comamonadaceae*, *Gallionellaceae* and *Rhodobacteraceae*); and *Verrucomicrobiota* (15 OTUs including mainly *Verrucomicrobiales*). Finally, 50 OTUs, to the right of the plot and specific to the Bard spring, are correlated with a high concentration of major elements (Mg^2+^, K^+^, Br^−^, HCO_3_^−^, Na^+^, Cl^−^), trace elements (Ba, Cu, Rb, Ni), TOC, H tot, C tot, as well as higher temperature, conductivity and pH measured in this spring (Figure 5A). Amongst these 50 OTUs, 7 belong to the *Acidobacteriota* (*Bryobacterales, Acidobacteriales*); 12 to the *Actinobacteriota* (*Acidothermus*, *Mycobacterium, Marmoricola, Conexibacter, Jatrophihabitans*); 8 to the *Bacteroidota* (*Sphingobacteriales, Bacteroidales, Cytophagales, Chitinophagales*) and 13 to the Proteobacteria (*Thiotrichaceae, Rhizobiales, Caulobacteraceae, Hyphomonadaceae*).

For the 2020 campaign, 93 OTUs were clustered together at the top right of the correlation circle (Figure 5B). These OTUs, mainly specific to the Dourioux source, are correlated with high concentrations of U, Cu, NO_3_^−^, ^222^Rn and Th, total N and PO_4_^3−^ and mainly low concentrations of major ions (HCO_3_^−^, Ca^2+^, Sr (water and sediment), Li^2+^, Na^+^, Mg^2+^, K^+^, Br^−^, Cl^−^, NH_4_^+^), low temperature, low conductivity and low pH (Figure 5B). Amongst these OTUs, affiliated to 15 different phyla, 10 belong to the *Acidobacteriota* (*Vicinamibacteria, Holophagae*); 12 to *Actinobacteriota* (*Streptomyces* sp.*, Rhodococcus* sp.*, Gaiella* sp.*, Arthrobacter* sp.*, and Microbacteriaceae*); 10 with *Bacteroidota* (*Chitinophagaceae, Ignavibacteriaceae, Microscillaceae*); 1 with *Bellovibrionota* (*Bacteriovoracaceae*); 8 OTUs with *Chloroflexi* (*Anaerolineaceae*); 1 with *Desulfobacterota* (*Geobacteraceae*); 9 OTUs with *Firmicutes* (*Bacillus* sp.*, Paenibacillus* sp.*, Sporosarcina* sp.*, Pelosinus* sp.); 5 OTUs with *Nitrospirota* (*Nitrospira*) and 21 OTUs with *Proteobacteria* (*Burkholderiales: Nitrosomonadaceae, Comamonadaceae, Sideroxydans* sp.*; Pseudomonadales; Rhizobiales: Hyphomicrobium* sp.*, Pseudolabrys* sp.). On the other hand, 5 OTUs present mainly in the Montagne spring (*Chryseolinea, Sphingobacteriales, Chloroflexi, unclassified Gammaproteobacteria* and *Xanthobacter*) formed a second cluster in the bottom right of Figure 5B. This OTUs are associated with high concentrations of ^210^Pb, Mn Ba (water + sediment), ^222^Rn, Th, Ba (water and sediment), Fe, As, and F^−^, but also with low concentrations of total N, PO_4_^3−^, Sr (water), Mg^2+^, Na^+^, Li^2+^, NH_4_^+^, K^+^, Br^−^, Cl^−^, low temperature, low conductivity and low pH.

To summarise, the structure of the bacterial communities in the Montagne and Dourioux springs, characterised by high concentrations of radioelements, are different from the other springs sampled due to the presence of OTUs specific to these two springs during the two sampling campaigns. Amongst these OTUs, several are positively correlated with high concentrations of radioelements (mainly U, ^222^Rn, Th), metals (Cu and Pb), nitrates, as well as low concentrations of major elements (oligotrophic condition). These OTUs are affiliated to *Acidobacteriota* (*Vicinamibacterales, Holophagae*); *Actinobacteriota* (*Arthrobacter* sp., *Streptomyces* sp., *Rhodococcus* sp.*, Gaiella* sp. *and Microbacteriaceae*); *Bacteroidota* (*Flavobacterium*, *Chitinophagaceae, Ignavibacteriaceae and Microscillaceae*); *Bellovibrionota* (*Bacteriovoracaceae*); *Chloroflexi* (*Anaerolineaceae*)*; Firmicutes* (*Paenibacillus, Bacillus, Pelosinus, Sporosarcina* sp.*, Desulfosporosinus and Fonticella*); *Nitrospirota* (*Nitrospira*); *Planctomycetota* (*Pirellulaceae*); *Proteobacteria* (*Burkholderiales, Nitrosomonadaceae, Comamonadaceae, Sideroxydans* sp.*, Pseudomonadales, Hyphomicrobium* sp.*, Pseudolabrys* sp., *Methylophilaceae, Xanthobacteraceae and Rhodocyclaceae*) and *Verrucomicrobiota* (*Chthoniobacteraceae*).

### 3.4 Difference in springs bacterial communities according to season

Comparison of the diversity indices obtained for each sample (Table 2) showed that only the mean Shannon diversity index measured for the Dourioux spring in October 2019 (5.71 ± 0.01) was significantly different from that measured in June 2020 (5.86 ± 0.04). For this same spring, the Chao1 richness index measured for the two campaigns was also significantly different (658.43 ± 34.59 and 732.06 ± 16.68 respectively). In addition, the Chao1 richness index measured for the Montagne spring was also significantly different between the two sampling campaigns (877.26 ± 26.04 and 768.04 ± 24.34 respectively). Therefore, for Dourioux and Montagne springs, the bacterial diversity differed according to the season.

**Figure 6:**
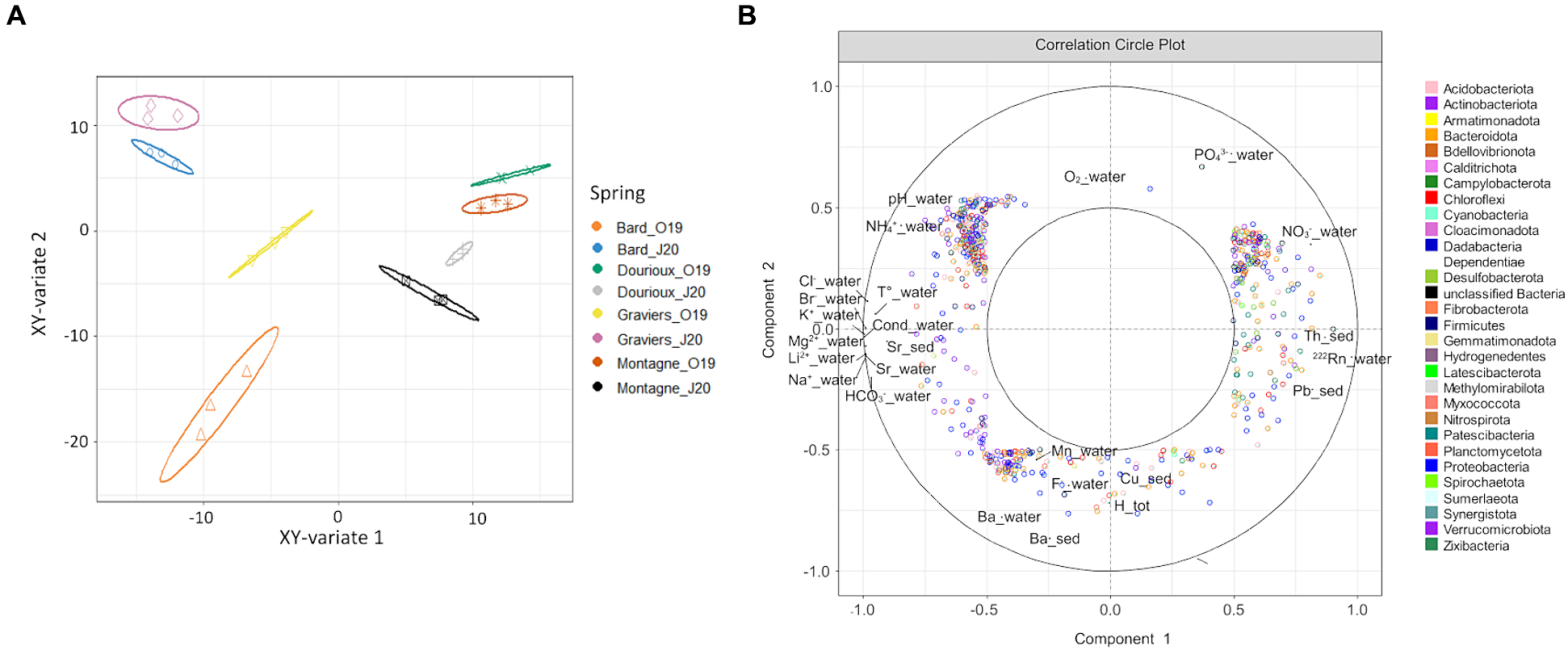
**(A)** Projection of source samples, in October 2019 and June 2020, into the space defined by the average components of the OTUs dataset and the physico-chemical parameters dataset, with ellipse plots at the 95% confidence level; **(B)** Correlation circle plot of sPLS analysis highlighting OTUs and physico-chemical parameters explaining the distribution of spring samples in the two seasons studied.

Subsequently, a sPLS analysis was conducted using the pre-filtered OTU table (1,469 OTUs) and physicochemical parameters (41) measured for the 4 springs sampled in October 2019 and June 2020 (Figure 6). Figure 6A shows the projection of the samples in the space defined by the mean components of the two data sets. It was observed that the four studied springs are separated according to their sampling period (Figure 6A). Figure 6B shows the variables that are responsible for the spatial distribution of the samples. In particular, the distribution of samples from the Bard and Graviers springs (Figure 6B) is explained by the presence of OTUs specific to these two springs, which are correlated with high concentrations of major ions (mainly HCO_3_^−^, Li^2+^, Mg^2+^, Br^−^, Sr, Cl^−^ and the conductivity on the left of the circle). On the other hand, the Montagne and Dourioux springs are distinguished from the Bard and Graviers springs (Figure 6B) by the presence of OTUs correlated with high concentrations of ^222^Rn, Th, U and NO_3_^−^ (right of the circle). However, our tests did not highlight which physicochemical parameters were related to the seasonal effect, as none of them explained the distribution of the 2020 samples compared to the 2019 samples (Figure 6B).

## 4 Discussion

This study is one of the first to examine bacterial communities exposed to long-term chronic irradiation in naturally radioactive mineral springs to evaluate the influence of radioelements on these communities. Employing a multidisciplinary approach, the study characterised five naturally radioactive mineral springs during two different seasons, considering both physico-chemical and biological (bacterial community) parameters. We hypothesised that the very long-term exposure of bacterial communities to the radionuclides present in these springs led to the selection of certain resistant bacterial populations, allowing the establishment of a community tolerant to these particular conditions.

### 4.1 Physico-chemical profiles of the studied springs

In order to determine whether the presence of radioelements or other environmental stress factors have an impact on the bacterial communities present, it seemed important to us to characterise the physico-chemical profiles of these springs as precisely as possible. Our results demonstrate that the springs studied have different physicochemical profiles and are discriminated into 2 clusters with Graviers, Bard and 3 Sauts on one side and Montagne and Dourioux on the other (Figure 2). Natural springs are influenced by their deep underground circulation and their chemical composition depends mainly on water-rock interactions. Different processes take place during the ascent of deep waters, such as dissolution, ion exchange, precipitation, leading to a modification of the chemical composition. Finally, most often, natural springs are mixtures of thermal water and surface water, the latter being poor in minerals and leading to a dilution of the deep water (Risler, 1974; Beaucaire et al., 1987; Négrel et al., 2000; Beauger et al., 2022). In particular, Bard, Graviers and 3 Sauts are mineral springs (Risler, 1974; Fouillac, 1983) characterised by high concentrations of major ions (Li^2+^, Na^+^, Cl^−^, HCO_3_^−^, K^+^, Mg^2+^, Br^−^, Ca^2+^) and trace elements (Sr) (Fouillac, 1983; Beaucaire et al., 1987; Négrel et al., 2000; Cornu et al., 2001; Beauger et al., 2018). On the other hand, Montagne and Dourioux springs differ from the other springs by the low concentration of major ions.

These two springs are also different from the other springs by high activities/concentrations of radioelements (principally ^222^Rn, Th, U) and high concentration of nitrates. These springs are located in uranium-rich granite regions. Indeed, the presence of torbernite (a mineral composed of hydrated uranyl phosphate) has been observed in quartz near the hamlet of Dourioux, highlighting numerous radioactive zones in this region (Alsac et al., 1988). Similarly, Montagne spring is located 3.75 km from a former uranium mine, which confirms the presence of uranium veins around this area (Millan et al., 2019). The uranium would be released during the ascent of the water to the emergence. Indeed, the slow cooling of the water in the cracks of the granite would favour the dissolution of uranium in the water (Beaucaire et al., 1987). Consequently, these springs are highly enriched in uranium and thorium compared to the other springs studied. These two springs were also characterised by the highest radon activities (average of 3,481 and 3,599 Bq/L for Dourioux and Montagne respectively, for both season). These values are much higher than the geometric mean of measured radon concentrations in groundwater and spring water compiled from the literature (29.1 Bq/L from 1,595 entries; Girault et al., 2018) but in the range of ^222^Rn concentrations measured in the most radioactive hydrothermal springs in the Mahallat region of Iran (145 to 2,731 Bq/L; Sohrabi, 1998). The Montagne spring is known to be one of the most radioactive in France, with a documented radon activity of 4,958 Bq/L (Risler, 1974). The water from these springs passing through these uranium-rich rocks will consequently become loaded with radon, which comes from successive radioactive decays of the ^238^U decay chain and facilitate its transfer from the subsurface to the surface (Horváth et al., 2000; Fonollosa et al., 2016; Ielsch et al., 2017; Girault et al., 2018).

A higher concentration of nitrates in the springs of Montagne and Dourioux, which is an anthropogenic indicator linked to their use in agriculture as fertiliser (Sebilo et al., 2013), shows that Montagne and Dourioux, located in an agricultural area, are affected by precipitations on their whatershed that mix with the spring water (Millan et al., 2019; Baker et al., 2022). This runoff could also explain decrease in major elements and conductivity observed in Montagne spring in 2019 due to dilution phenomena. In conclusion, according to the observations made on the springs studied during the two campaigns, these springs have different physicochemical profiles and represent distinct biotopes, including two which are distinguished by high concentrations/activities of radioelements (Montagne and Dourioux).

### 4.2 Relationship between the different environmental parameters and bacterial communities in mineral spring sediments: what is the impact of radioactivity compared to other parameters?

Studying the bacterial communities in these naturally radioactive mineral springs provides an opportunity to study how these communities are adapted to these naturally radioactive conditions. The presence of radioelements in these sources could lead to selection pressure on the bacterial communities in these sources, due to both chemotoxic and radiotoxic effects. In particular, radiation exposure can have an impact on the communities of microorganisms present in these springs (Kolovi et al., 2023). Although the presence of radioelements can lead to a decrease in bacterial diversity (Suriya et al., 2017; Hoyos-Hernandez et al., 2019; Rogiers et al., 2021), research typically shows no clear associations between the presence of radioelements and bacterial diversity (Radeva et al., 2013; Sitte et al., 2015; Theodorakopoulos et al., 2017; Li et al., 2018; Zeng et al., 2020). Our results show that the presence of radionuclides did not lead to a decrease in bacterial diversity and richness in the Montagne and Dourioux springs (characterised by higher radon activity and radioelement concentrations—Supplementary Tables S1A,B) compared to the other springs for both sampling periods. In these naturally radioactive mineral springs, the communities have been exposed to radioelements for thousands of years. It is therefore conceivable that these bacterial communities have adapted to these conditions, leading to a high level of richness and diversity in the Dourioux and Montagne springs. Indeed, radiation resistant and tolerant bacteria might be able to modify the geochemical conditions in the spring sediments, allowing other bacteria with different metabolisms to grow using the substrates released by these resistant bacterial populations. In addition, radioactivity is only one of many abiotic factors that can influence the bacterial diversity. Other environmental parameters can directly influence bacterial communities (presence of toxic metals, lack of nutrients, different temperature or pH; Lauber et al., 2008), but also play a role in the speciation of certain radioelements (modification of pH, complexation with organic matter, iron oxide or manganese) thus impacting their mobility, bioavailability and consequently their toxicity on bacterial communities (Cumberland et al., 2016; Husson et al., 2019). Therefore, it was important to discriminate the effects of the different parameters, in order to observe if radioactivity in these springs could explain a distinctive bacterial composition with the presence of specific OTUs, and if these OTUs could play a role in the biogeochemistry of these radioelements.

In particular, the present study determined that specific identified OTUs *Actinobacteriota* (*Microbacteriaceae, Streptomyces, Arthrobacter, Gaiella* and *Rhodococcus*), *Acidobacteriota* (*Holophagae*), *Bacteroidetes* (*Chitinophagaceae*), C*hloroflexi* (*Anaerolineaceae*), *Desulfobacterota* (*Geobacteraceae*), *Proteobacteria* (*Pseudolabrys*, *Pseudomonadales, Burkholderiales, Comamonadaceae*) and *Firmicutes* (*Bacillus*, *Desulfosporosinus, Paenibacillus, Fonticella, Sporosarcina* et *Pelosinus*) which predominates in the highest radioactive springs (Montagne and Dourioux) were found to be significantly correlated with high concentrations of radioelements (mainly U, ^222^Rn, Th), metals (Cu and Pb), nitrates, as well as low concentrations of major elements (oligotrophic condition) (Figure 5). These OTUs could have a particular metabolism that allows them to resist the toxicity of these metals/radioelements and to thrive in these extreme environments as has been shown in various other ecosystems (Mondani et al., 2011; Carvajal et al., 2012; Chapon et al., 2012; Theodorakopoulos et al., 2015; Islam and Sar, 2016; Lusa et al., 2019; Sutcliffe et al., 2019; Tang et al., 2021; Brzoska et al., 2022; Yang et al., 2022). For example, *Gaiella* and *Rhodococcus* have demonstrated their ability to grow in heavy metal contaminated river sediments (Yang et al., 2022), as have *Microbacteriaceae*, *Arthrobacter*, *Pseudomonadales*, *Burkholderiales*, and *Streptomyces*, which have been observed in environments with high radiation or radioelement concentrations (Carvajal et al., 2012; Chapon et al., 2012; Islam and Sar, 2016; Lusa et al., 2019; Tang et al., 2021). These specific OTUs able to grow in these metal and radioelement rich springs could interact with these elements resulting in immobilisation and a reduction in their availability (Ryzhkov et al., 2007; Simonoff et al., 2007; Merroun and Selenska-Pobell, 2008; Acharya, 2015; Thorgersen et al., 2017; Kolhe et al., 2018). Bacteria interact with metals and radioelements through various mechanisms, including biotransformation (oxidation or reduction of metals and radioelements by bacterial activities), bioaccumulation (absorption and accumulation by cells), biosorption (passive adsorption by complexation with proteins, polysaccharides and microbial biomolecules) and biomineralisation (precipitation of metals or radioelements by ligands produced enzymatically by bacteria) (Merroun and Selenska-Pobell, 2008; Kolhe et al., 2018). In particular, members of *Microbacteriaceae, Arthrobacter, Pseudomonadales, Chitinophagaceae*, *Bacillus, Paenibacillus, Streptomyces*, *Pelosinus* and *Sporosarcina* could reduce the toxicity of uranium through different processes, such as: precipitation of uranium in a stable and insoluble mineral form via constitutive phosphatase activity (*Bacillus, Paenibacillus, Pseudomonadales*) (Beazley et al., 2007; Martinez et al., 2007; Choudhary and Sar, 2011; Reitz et al., 2014; Zhang et al., 2018); intracellular accumulation of uranium as a precipitate [*Bacillus, Arthrobacter, Microbacteriaceae* (Suzuki and Banfield, 2004; Li et al., 2014; Theodorakopoulos et al., 2015)]; or uranium complexation adsorption with cell surface functional groups [*Streptomyces, Bacillus* (Li et al., 2014, 2016)], surface S-layer proteins [*Pelosinus and Sporosarcina* (Ryzhkov et al., 2007; Thorgersen et al., 2017)] and the lipopolysaccharide layer [*Chitinophagaceae* (Brzoska et al., 2022)]. The presence of these OTUs, correlated with the high concentrations of metals (Cu and Pb) and radioelements (^222^Rn, U and Th) in the springs of Montagne and Dourioux, is very interesting because they could interact with these elements leading to a reduction in their solubility and bioavailability.

On the other hand, OTUs affiliated to *Desulfosporosinus, Geobacteraceae, Comamonadaceae* and *Holophagae* present in the springs of Montagne and Dourioux, demonstrate the presence of sulphate-reducing and iron-reducing bacteria which could be involved in the bioreduction of metals and radioelements such as uranium. The sediments of these carbo-gaseous mineral springs are favourable to anoxic conditions allowing the development of anaerobic bacteria such as sulphate-reducing and iron-reducing bacteria. Therefore, these OTUs could play an important role in the reduction reactions of uranium and other metals in these mineral springs, leading to their immobilisation (Lloyd, 2003; Nevin et al., 2003; Suzuki et al., 2004; Cardenas et al., 2008; Gihring et al., 2011; Mondani et al., 2011; Sitte et al., 2015; Sutcliffe et al., 2017). In addition to metal-reducing species, fermentative bacteria, such as *Anaerolineaceae*, associated with high uranium concentrations in these two springs, are also favoured by the anoxic conditions present in these springs. Given the potential production of organic acids (such as acetate) by *Anaerolineaceae* (Jroundi et al., 2020) and their high abundance in the sediments of these springs, syntrophic relationships may occur between these fermentative bacteria and metal-reducing bacteria (Sutcliffe et al., 2017). Indeed, the acetate released by these fermentative bacteria could be used as an electron donor and/or carbon source by the metal-reducing bacteria in support of their growth.

Apart from elevated concentrations of radionuclides and metals, Montagne and Dourioux springs exhibited high nitrate concentrations. These compounds support the presence of OTUs involved in nitrification (*Nitrospira*, *Nitrosomonadaceae*) and denitrification (*Hyphomicrobium*), important processes in the nitrogen cycle (Martineau et al., 2013; Pester et al., 2014). Additionally, it has been shown that in aquifers co-contaminated with uranium and nitrate, denitrifiers play a key role in the bioremediation of these ecosystems, as nitrates can prevent the reduction of U(VI) by competing with it as an electron acceptor (Yan et al., 2003; Spain and Krumholz, 2012).

The co-relationships between OTUs associated with ferric or sulphate reducing species, fermentative species and denitrifying species, with high metal/radioelement concentrations reflect associations of bacteria fulfilling complementary functions allowing their growth in these particular environments. Indeed, the bioreduction of uranium by ferric/sulphate reducing bacteria could lead to a decrease in the bioavailability and, consequently, the toxicity of uranium, favouring the growth of fermentative and denitrifying bacteria. On the other hand, this bioreduction would be favoured both by the contribution of organic carbon sources by the fermentative bacteria and the reduction of the nitrate concentration by the denitrifying bacteria. Finally, the supply of organic carbon sources by the fermenting bacteria for the denitrifying bacteria would also favour denitrification, and thus the bioreduction of uranium.

To summarise, the springs of Dourioux and Montagne are characterised by distinct populations positively linked to the radioelements (U, Th and ^222^Rn) measured in these two springs. This suggests that the presence of these radioelements could have led to a selection of bacterial species capable of developing in these extreme conditions. Several OTUs specific to these two springs could be involved in the biogeochemistry of metals and radioelements, leading to a reduction in their solubility and bioavailability, which would allow other bacterial species sensitive to these metals/radioelements to develop. In particular, co-correlations between ferric/sulphate reducing bacteria (capable of bioreducing uranium) and fermentative bacteria (releasing carbon sources), reflect associations of bacteria fulfilling complementary functions that allow them to grow in these particular environments. Consequently, these commensal or mutualistic relationships could maintain diversity in these extreme environments. However, our results show that the presence of certain OTUs is not associated with a single compound but rather a sum of co-correlations with various physicochemical parameters. As microbes are never confronted with a single contaminant in the field, it is difficult to differentiate the effects of a single metal species on microbial communities *in situ* (Sitte et al., 2015; Rogiers et al., 2021). Further studies aimed at determining the (bio)available fraction of radioelements/metals would enable better discrimination of the effects of these elements on bacterial communities. In addition, future work using metagenomic analysis of overall bacterial functions will make it possible to study the metagenome of these samples and describe their overall functioning.

### 4.3 Seasonal variation of bacterial communities

The last point of our study was to determine if the bacterial communities change with the seasons in these mineral springs, which are insular aquatic systems fed by groundwater that is generally stable over time (Négrel et al., 2000; Cornu et al., 2001). Nevertheless, our results show that the physico-chemical parameters of some springs have changed between the two sampling periods. In particular, variations in physico-chemical parameters due to dilution phenomena caused by runoff linked to precipitation have been observed in Montagne and Dourioux springs. As bacterial communities are related to concentration levels and/or different geochemical and physiological environmental conditions (Radeva et al., 2013; Sowmya et al., 2020; Lopez-Fernandez et al., 2021), these variations can explain the significant differences in alpha diversity (diversity and richness) measured in Dourioux and Montagne springs between the two sampling periods (Table 2). Furthermore, the structure of the bacterial communities changed according to the sampling season (Figure 6A). However, any effect of the season could be highlighted by the physico-chemical parameters measured (Figure 6B). Indeed, the physico-chemical parameters used for this analysis were better at discriminating the springs than explaining the observed differences due to the season. These springs contain settled bacterial populations resulting from the selection pressure associated with different physico-chemical parameters (major ions, radioelements, etc.) specific to each spring. Nevertheless, we hypothesise that changes in nutrient supply could occur in these springs depending on the season (leaf supply, nitrates, phytoplankton, cyanobacteria) and could lead to changes in bacterial populations within each spring. Future work could include taking a larger number of samples from the same spring each season to obtain more statistics to discriminate the parameters that explain the evolution of bacterial communities according to season.

## 5 Conclusion

In summary, the aim of this study was to determine whether radionuclides could be drivers of the structure of bacterial communities in natural radioactive mineral springs. Indeed, these mineral springs are peculiar environments where radioactivity could lead to the selection of certain resistant bacterial populations allowing the establishment of tolerant populations and, consequently, to a specific bacterial community adapted to these conditions. Our results indicate that these springs represent distinct biotopes and contain bacterial populations specific to each one, which change according to the seasons, linked to the physicochemical parameters specific to each one. In particular, the two springs (Dourioux and Montagne) with high activity/concentration of radioelements were characterised by very distinct populations compared to the other springs studied. It is interesting to note that in these springs strong correlations were observed between the radioelements (U, Th and ^222^Rn) and their specific OTUs. Several of these specific OTUs, such as the ferric- or sulphate-reducing bacteria identified, could be involved in the biogeochemistry of the metals and radionuclides present in these springs, through bioreduction for example, which would reduce the solubility of these elements, and consequently their bioavailability and toxicity. Hence, other bacterial species, sensitive to these toxic metals/radioelements, could still develop in these extreme environments, such as fermentative bacteria or denitrifying bacteria, which are also specific to these two springs. On the other hand, these fermentative and denitrifying bacteria could promote the bioreduction of radioelements by iron/sulphate-reducing bacteria, either by releasing sources of organic carbon or by reducing the nitrate concentrations. The associations of specific bacteria in the Dourioux and Montagne springs, which perform complementary functions that allow their growth in these particular environments, could explain how diversity and richness are maintained in these extreme environments. In conclusion, this study suggests that radioelements are partly drivers of bacterial community structure, particularly in these two mineral springs with high levels of radioactivity. However, the high complexity of the bacterial communities identified in these different springs requires in-depth research into their different functions to understand how these communities co-evolve in these extreme environments.

## Data availability statement

The datasets presented in this study can be found in online repositories. The names of the repository/repositories and accession number(s) can be found in the article/Supplementary material.

## Author contributions

GH: Conceptualization, Formal analysis, Investigation, Visualization, Writing – original draft, Writing – review & editing. CS: Funding acquisition, Investigation, Supervision, Writing – original draft, Writing – review & editing. CB: Investigation, Resources, Writing – original draft, Writing – review & editing. AB: Resources, Writing – review & editing. VB: Investigation, Resources, Writing – original draft, Writing – review & editing. PC: Resources, Writing – review & editing. GM: Resources, Writing – review & editing. M-HV: Resources, Writing – review & editing. CM: Conceptualization, Investigation, Resources, Supervision, Writing – original draft, Writing – review & editing.

## Footnotes

^1^ https://www.r-project.org/
