# Supplementary material for: Radioactivity as a driver of bacterial community composition in naturally radioactive mineral springs in the French Massif Central
Source: Front Microbiol. 2024 Jul 23;15:1423342. doi: 10.3389/fmicb.2024.1423342 (PMC11300270; doi:10.3389/fmicb.2024.1423342)
Supplement: Supplementary file 1 [file Data_Sheet_1.docx]

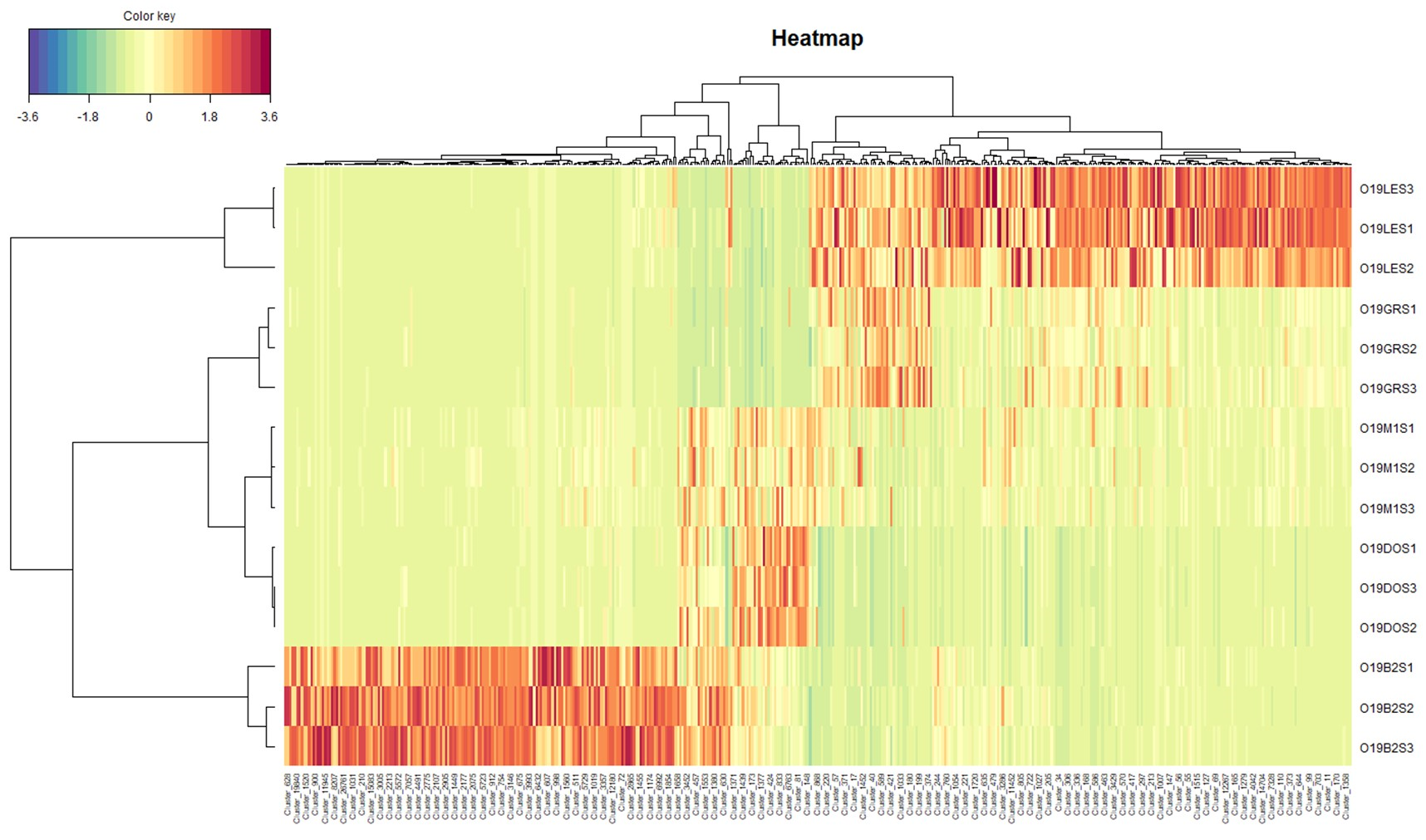


Figure S1 : Clustered Image Map representing the relative abundance of the OTUs selected in each sample by sPLS-DA on the OTUs pre-filtered data across the first two components in October 2019. The identification of the sample is given in the right hand side of the plot, where the digits indicates the Sampling date (O19: October 2019), following by the spring name (LE: 3 Sauts, GR: Graviers, M1: Montagne, DO: Dourioux, B2: Bard), sample matrix (S: sediment) and replicate number respectively (n=3). Further information related to each cluster can be found in the OTU table (supplementary data).


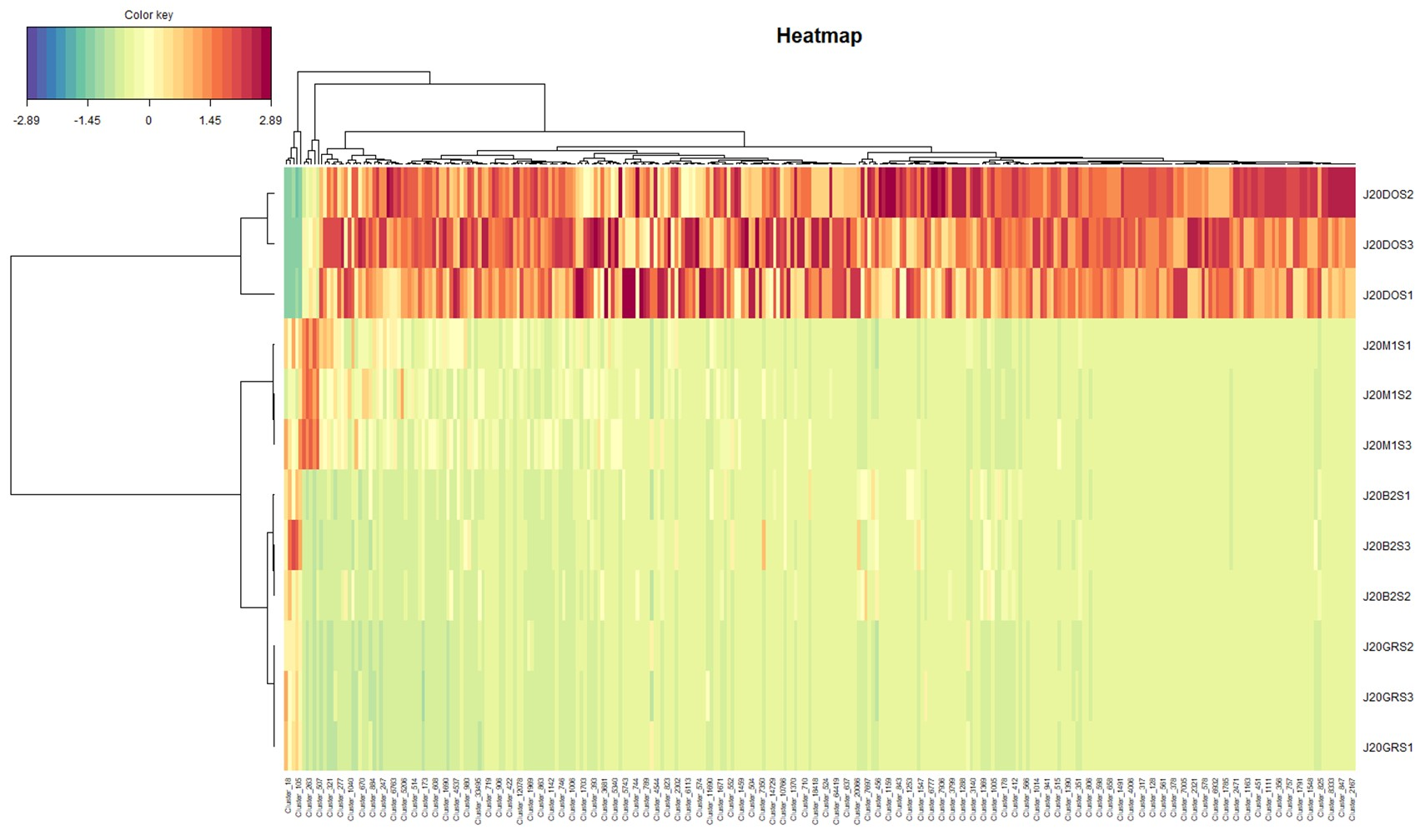


Figure S2 : Clustered Image Map representing the relative abundance of the OTUs selected in each sample by sPLS-DA on the OTUs pre-filtered data across the first two components in June 2020. The identification of the sample is given in the right hand side of the plot, where the digits indicates the Sampling date (J20: June 2020),), following by the spring name (DO: Dourioux, , M1: Montagne, B2: Bard, GR: Graviers), sample matrix (S: sediment) and replicate number respectively (n=3). Further information related to each cluster can be found in the OTU table (supplementary data).

Table S1.A : Concentrations of TOC (g(C)/Kg), total CHNS (g/kg), trace metals (µg/g except for Fe in mg/g), and radioelement activity measured in sediment samples for each site during the October 2019 and June 2020 campaigns

| Springs | Sampling | Mn (µg/g) | Cu (µg/g) | As (µg/g) | Rb (µg/g) | Sr (µg/g) | Ba (µg/g) | Pb (µg/g) | Th (µg/g) | U (µg/g) | Cr (µg/g) | Ni (µg/g) | Fe (mg/g) | ^226^Ra (Bq/g) | ^210^Pb (Bq/g) | ^228^Ac (Bq/g) | ^212^Pb (Bq/g) | TOC (g(C)/kg) | N tot (g/kg) | H tot (g/kg) | S tot (g/kg) | C tot (g/kg) |
| --- | --- | --- | --- | --- | --- | --- | --- | --- | --- | --- | --- | --- | --- | --- | --- | --- | --- | --- | --- | --- | --- | --- |
| Bard | October 2019 | 333 | 40.47 | 310 | 111.72 | 795. | 1316 | 45.80 | 4.48 | 0.73 | 42.67 | 29.18 | 84.62 | 2.19 | 1.25 | 0.67 | 1.10 | 193 | 10.53 | 31.59 | 5.16 | 226 |
|  | June 2020 | 246 | 12.20 | 25 | 111.54 | 491 | 465 | 22.16 | 1.94 | 0.68 | N.A | N.A | 26.61 | 0.21 | 0.11 | 0.19 | 0.17 | 172 | 9.36 | 24.53 | 3.36 | 180 |
| Dourioux | October 2019 | 571 | 0.07 | 103 | 65.77 | 63 | 96 | 72.25 | 9.67 | 16.12 | 0.07 | 25.77 | 25.68 | 0.60 | 1.44 | 0.11 | 0.09 | 78 | 10.75 | 21.19 | 4.44 | 124 |
|  | June 2020 | 615 | 43.58 | 145 | 44.97 | 37 | 74 | 52.78 | 6.34 | 21.48 | N.A | N.A | 16.96 | 0.76 | 2.59 | 0.07 | 0.07 | 104 | 10.52 | 20.36 | 2.99 | 123 |
| Graviers | October 2019 | 391 | 5.64 | 5426 | 63.34 | 802 | 476 | 13.60 | 4.24 | 1.85 | 21.42 | 10.56 | 123.06 | 26.73 | 3.15 | 17.97 | 9.77 | 53 | 8.54 | 17.19 | 12.55 | 111 |
|  | June 2020 | 415 | 4.94 | 2443 | 36.62 | 874 | 279 | 10.65 | 2.21 | 1.48 | N.A | N.A | 90.51 | 25.47 | 2.50 | 10.59 | 4.98 | 61 | 6.62 | 13.38 | 8.13 | 123 |
| 3 Sauts | October 2019 | 530 | 0.02 | 40599 | 31.80 | 896 | 429 | 6.52 | 0.33 | 0.23 | 0.01 | 1.83 | 165.27 | 2.08 | 2.94 | 1.14 | 0.66 | 87 | 7.57 | 17.24 | 5.15 | 147 |
|  | June 2020 | N.A | N.A | N.A | N.A | N.A | N.A | N.A | N.A | N.A | N.A | N.A | N.A | N.A | N.A | N.A | N.A | N.A | N.A | N.A | N.A | N.A |
| Montagne | October 2019 | 1251 | 13.25 | 2849 | 48.34 | 233 | 627 | 111.82 | 11.70 | 5.33 | 19.26 | 7.71 | 113.29 | 8.61 | 8.06 | 0.84 | 0.56 | 93 | 8.92 | 24.15 | 3.88 | 205 |
|  | June 2020 | 1413 | 13.15 | 3415 | 21.57 | 341 | 1145 | 73.99 | 7.00 | 5.95 | N.A | N.A | 222.98 | 20.56 | 6.39 | 2.03 | 0.38 | 27 | 1.32 | 22.11 | 1.70 | 60 |

Table S1.B : Physical variables (conductivity, temperature, dissolved oxygen, pH) and chemical variables (major ions, trace metals and radioelements) measured in water samples for each site during the October 2019 and June 2020 campaigns

| Springs | Sampling | ^222^Rn (Bq/L) | Mn (µg/L) | Sr (µg/L) | Ba (µg/L) | Li^2+^ (mg/L) | Na^+^ (mg/L) | NH_4_^+^ (mg/L) | K^+^ (mg/L) | Mg^2+^ (mg/L) | Ca^2+^ (mg/L) | F^-^ (mg/L) | Cl^-^ (mg/L) | NO_2_^-^ (mg/L) | Br^-^ (mg/L) | NO_3_^-^ (mg/L) | PO_4_^3-^ (mg/L) | SO_4_^2^- (mg/L) | HCO_3_^-^ (mg/L) | Conductivity (µS/cm) | pH | O_2_ (%) | T (°C) |
| --- | --- | --- | --- | --- | --- | --- | --- | --- | --- | --- | --- | --- | --- | --- | --- | --- | --- | --- | --- | --- | --- | --- | --- |
| Bard | October 2019 | 10 | 508 | 4090 | 278 | 5.20 | 1028.88 | 0.47 | 185.88 | 136.02 | 233.76 | 0.87 | 702.07 | 0.02 | 1.88 | 0.21 | 0.12 | 34.03 | 3012 | 6380 | 6.81 | 3.1 | 14.7 |
|  | June 2020 | 5 | 463 | 3880 | 210 | 4.98 | 974.68 | 1.15 | 184.06 | 126.87 | 223.32 | 0.80 | 680.38 | 0.02 | 1.71 | 0.12 | 0.19 | 30.25 | 2810 | 6180 | 7.22 | 2.9 | 15.5 |
| Dourioux | October 2019 | 3110 | 12 | 173 | 20 | 0.02 | 12.28 | 0.01 | 4.47 | 7.67 | 21.63 | 0.10 | 16.53 | 0.02 | 0.02 | 17.74 | 0.57 | 17.47 | 85 | 255 | 6.73 | 10.1 | 10.7 |
|  | June 2020 | 3852 | 11 | 180 | 13 | 0.01 | 12.32 | 0.01 | 9.19 | 8.43 | 24.77 | 0.12 | 12.89 | 0.01 | 0.01 | 12.21 | 0.43 | 12.85 | 130 | 246 | 6.35 | 22.9 | 10.7 |
| Graviers | October 2019 | 38 | 518 | 3360 | 68 | 4.16 | 823.10 | 0.67 | 159.59 | 102.66 | 381.87 | 0.64 | 739.44 | 0.02 | 1.66 | 0.63 | 0.13 | 134.40 | 2730 | 5430 | 6.69 | 19.5 | 14.4 |
|  | June 2020 | 29 | 520 | 3410 | 68 | 4.12 | 808.63 | 1.63 | 162.59 | 111.50 | 424.36 | 0.60 | 792.79 | 0.03 | 1.65 | 0.33 | 0.37 | 124.42 | 2510 | 6360 | 7.48 | 30.8 | 15.9 |
| 3 Sauts | October 2019 | 322 | 426 | 1660 | 33 | 5.41 | 803.89 | 0.06 | 69.23 | 39.31 | 135.35 | 0.86 | 319.64 | 0.01 | 0.89 | 0.71 | 0.06 | 178.05 | 1940 | 3890 | 6.66 | 12.8 | 13.1 |
|  | June 2020 | N.A | N.A | N.A | N.A | N.A | N.A | N.A | N.A | N.A | N.A | N.A | N.A | N.A | N.A | N.A | N.A | N.A | N.A | N.A | N.A | N.A | - |
| Montagne | October 2019 | 3453 | 47 | 296 | 47 | 0.24 | 56.21 | 0.06 | 5.15 | 5.49 | 67.46 | 0.86 | 3.48 | 0.03 | 0.06 | 17.66 | 0.30 | 8.40 | 340 | 693 | 6.54 | 6.8 | 12.5 |
|  | June 2020 | 3746 | 1296 | 1070 | 260 | 1.00 | 182.31 | 0.02 | 18.85 | 21.26 | 261.04 | 1.60 | 3.70 | 0.02 | 0.04 | 0.05 | 0.07 | 13.30 | 1400 | 1702 | 6.32 | 4.6 | 12.2 |
